# Open Practices and Resources for Collaborative Digital Pathology

**DOI:** 10.3389/fmed.2019.00255

**Published:** 2019-11-14

**Authors:** Raphaël Marée

**Affiliations:** Montefiore Institute, University of Liège, Liège, Belgium

**Keywords:** digital pathology, open source, open data, collaboration, open science, image analysis

## Abstract

In this paper, we describe open practices and open resources in the field of digital pathology with a specific focus on approaches that ease collaboration in research and education settings. Our review includes open access journals and open peer review, open-source software (libraries, desktop tools, and web applications), and open access collections. We illustrate applications and discuss current limitations and perspectives.

## Introduction

Open science paradigm promotes open, reproducible science through the sharing of all processes, data, material, and methods. The underlying philosophy is that everyone's work should improve knowledge by enabling shared resources by all without any barrier. The main pillars of open science are open access (OA) and open peer review (OPR), open-source software development (OSS), open-source hardware (OSH), open data (OD), and open educational resources (OER). OA and OPR can be applied to all forms of published research output (academic journal articles, conference papers, book chapters, etc.). OSS relates to a family of computer tool in which source code (its underlying recipe written using a human-readable programming language) is disclosed. This contrasts with the closed (proprietary) paradigm where each entity (research lab or company) can only rely on their own developments and are not able to study other's works. Similarly, OSH involves the development of physical systems (e.g., microscopes) through the use of publicly shared design information. OD is data (textual or non-textual) that anyone can access, use, reuse, or distribute. It can be materialized in the form of free access databases as well as free digital file formats (a published specification to describe how information is encoded in a computer file). OER encompasses freely accessible digital documents (textbooks, presentation slides, samples, etc.) that are useful for teaching and learning. In addition to impact on science, medicine, and education, the open science paradigm leads to the development of novel interactions and business models in the industry such as open collaboration and open innovation. Interestingly, the open philosophy also contributes to overcome vendor lock-in situations where a customer using a proprietary product or service cannot easily transition to another technology due to incompatibilities, inefficient processes, or contract constraints.

In this Mini-Review paper, we discuss open initiatives that might pave the way to more collaborative digital pathology (DP) in the future.

## Open Practices and Resources

In this section, we briefly list the open practices and resources we are aware of at the time of submission (in early summer 2019) in the field of DP.

### OA Journals and OPR

The content of an OA journal is open to all, with no access fees. The *Journal of Pathology Informatics* (http://www.jpathinformatics.org/) is an OA peer-reviewed online journal dedicated to pathology informatics. It publishes all types of papers related to DP, e.g., development and empirical evaluation of computational algorithms; studies and use cases in clinical, research, or education settings; book reviews or literature surveys; scientific conference reports; etc. Articles are distributed under the terms of a Creative Commons license, which allows others to remix, tweak, and build upon the work non-commercially, as long as appropriate credit is given and the new creations are licensed under the identical terms. Interestingly, other scientific journals of wider scope in the field of biomedical research, medicine, or pathology follow the OA principle and accept papers in the field of DP. It includes Diagnostic Pathology (https://diagnosticpathology.biomedcentral.com/) that considers research in surgical and clinical pathology and also focuses on the technological aspects of pathology including virtual microscopy. Authors are the copyright holders of their article, and according to the BioMed Central license agreement they grant to any third party, in advance and in perpetuity, the right to use, reproduce, or disseminate their article. Similarly, the multidisciplinary and OA journal *Nature Scientific Reports* regularly publishes papers in the field of DP (https://www.nature.com/search?q=digital+pathology). Obviously, our Mini-Review paper in the Computational Pathology special issue of the Pathology section of *Frontiers of Medicine* (https://www.frontiersin.org/journals/medicine/sections/pathology) suggests that other OA journals will also provide the opportunity to publish papers in the DP field.

OPR is an emerging practice with various alternative review methods that seek to make the peer review process more transparent. Generally, scientific conferences implementing OPR mechanisms are still rather few, and well-known venues for DP [European Conference on Digital Pathology (ECDP), and the Computational Pathology Symposium at the European Conference of Pathology (ECP)] did not follow these principles so far. Notably, the reviews of the latest edition of the MICCAI workshop on Computational Pathology (COMPAY) were organized in a single-blind fashion and were made public along with the final version of the papers (https://openreview.net/group?id=MICCAI.org/2019/Workshop/COMPAY). Other conferences with a broader scope, such as MIDL (Medical Imaging with Deep Learning) and ICLR (International Conference on Learning Representations) have also published papers with some DP content after an open review evaluation process.

### OSS for Image Analysis and Collaborative Research

An OSS is a computer tool in which the source code (sets of operations written in plain text according to a computer language) is disclosed. It is released under a license in which the copyright holder grants users the rights to study, change, and distribute the software to anyone and for any purpose. A permissive license allows redistribution with minimal requirements (e.g., requiring little more than attributing the original portions of the licensed code to the original developers in future derivative works) while a copyleft license stipulates that the same rights have to be preserved in derivative works created later.

There is a long tradition of OSS in the bioinformatics and biomedical imaging communities. More than 30 years ago, NIH Image and ImageJ have been pioneers as open tools for the analysis of scientific images (1). In the DP field, the first open-source initiatives date back to the early 2000s. In the following, we present OSS according to their types: libraries, algorithms, user-friendly desktop tools for isolated image analysis, and user-friendly web-based tools for collaborative image analysis. At the time of submission, about fifty software repositories tagged with “digital pathology” can be found on the open-source Github repository (https://github.com/search?q=digital+pathology). In the following subsections, we list published software tools that have already had some impact or are promising for the field.

#### Libraries

Libraries are foundational tools that are not directly usable for end-users (e.g., pathologists or biomedical researchers) but that enable the development (by computer scientists) of a wide variety of downstream software. In particular, it includes tools to extract the imaging data from files generated by digital slide scanners, or to convert these into other image formats readable by existing software. The development of Openslide (https://openslide.org/, permissive license) was initiated at the Carnegie Mellon University. It enables to extract image data from proprietary DP slide scanner formats. Bioformats (https://www.openmicroscopy.org/bio-formats/, copyleft license) is a library developed by the Open Microscopy Environment consortium for reading and writing image data using standardized, open formats. As it was originally designed for low- or medium-spatial resolution microscopy images, in its early days, this library combined with an OMERO server (2) was not effective for processing high-resolution pathology images. Latest versions improve support of DP images and propose a new standardized, pyramidal, open image format (3). The DICOM-izer in Orthanc software [https://www.orthanc-server.com/static.php?page=wsi (4), copyleft license] is a stand-alone, cross-platform command-line tool to convert a whole-slide image (WSI) from a non-DICOM format to DICOM (following Supplement 145). It has to be noted that none of these libraries support all existing DP formats. For example, Bioformats does not explicitly support native slide scanners format from 3DHistech, Philips, and Sakura, while OpenSlide does, but OpenSlide does not support PerkinElmer Vectra format while Bioformats does. Therefore, currently available software (see below) have to combine these libraries to cover the wide range of available formats. Other open-source tools related to image handling have been developed previously (5, 6) but their use has remained rather limited so far as they were not neither maintained nor seamlessly integrated into widely applicable, usable software.

#### Algorithms and Image Analysis Packages

Image analysis packages are implementations of image analysis algorithms or workflows for object classification (e.g., cell types), region segmentation (e.g., tumor delineation), object counting (e.g., nucleus counting), or image quality control (e.g., artifacts detection). These packages are often based on foundational libraries including the aforementioned OpenSlide, scikit-learn (machine learning), Tensorflow, PyTorch or Keras (deep learning), or OpenCV (computer vision). They are often stand-alone command-line tools developed by computer scientists for computer scientists. SLDC (https://github.com/waliens/sldc, permissive license) is a framework for object detection and classification in multi-gigapixel images. HistoQC [https://github.com/choosehappy/HistoQC (7), permissive license] is a tool to perform quality control of digitized slides, e.g., delineating artifacts and discovering cohort-level outliers based on a combination of various image metrics. BIRL (https://github.com/Borda/BIRL, permissive license) is a software package for the benchmarking of whole-slide image registration methods. Other collection of scripts for stain/color normalization, focus quality assessment, and deep learning are available on Github as previously mentioned.

#### Desktop Applications for Image Analysis

Existing desktop software tool (i.e., running on an individual computer) for bioimage analysis such as ImageJ/FIJI [https://fiji.sc/, (1)], CellProfiler (https://cellprofiler.org/, permissive license), or Icy [http://icy.bioimageanalysis.org/ (8); copyleft license] can be used in DP studies [e.g., (9) used Icy for kidney tissue analysis]. Other specific tools were recently developed with features tailored for DP or to exploit more efficiently large image files. QuPath [https://qupath.github.io/ (10); copyleft license] was initiated at the Queen's University Belfast. It is a cross-platform, user-friendly, WSI viewer that incorporates various features for whole-slide images, in particular tissue microarrays, including dearraying, stain estimation, cell detection and feature computation, trainable cell classification, batch processing, and survival analysis. It can be complemented by image analysis routines written in ImageJ macro language, Matlab, or call machine learning algorithms from WEKA. ASAP is another desktop application (https://computationalpathologygroup.github.io/ASAP/, copyleft license) initiated at the Radboud University Medical Center. It can run on 64-bit Windows and Linux machines. It offers whole-slide image viewing and annotation functionalities, as well as mechanisms to overlay segmentation or likelihood maps produced by machine learning algorithms. Orbit (http://www.orbit.bio/ https://github.com/mstritt/, copyleft license) was initiated at Actelion Pharmaceuticals Ltd. It implements various algorithms for tissue quantification and it offers interfaces to connect to an OMERO image server.

#### Web-Based Applications for Collaborative Image Analysis

A web-based application is a program that is accessed over a network connection using the Hypertext Transfer Protocol (HTTP) in a web browser. In such a setting, processing operations (such as image analysis tasks) can be initiated by the end-user via the web interface but they are executed on servers (on the intranet, on the internet, or in the cloud).

Although there are several OSS web-based tools for the visualization of WSI (e.g., caMicroscope, http://camicroscope.org/, permissive license; Microdraw, http://microdraw.pasteur.fr/, copyleft license), to our knowledge, there are only two that also offer advanced features to work collaboratively, such as user and project management, and seamlessly integrated tools for semi-automated image analysis.

The first one, Cytomine [https://www.cytomine.org (11), permissive license], was initiated at the University of Liège in 2010. With Cytomine, multiple remote collaborators can organize their imaging data into projects with secured access. This tool relies on generic data models that enable to semantically delineate regions of interests in images in a standardized way (using ontologies and metadata), e.g., for histology, cytology, and other imaging modalities [e.g., multispectral; (12)] without restriction of application domain. It also provides mechanisms (RESTful API, Python and Java clients, container technologies) to readily proofread and share image quantifications (e.g., cell classification, tumor delineation, or cell counting) produced by any computer vision or machine/deep learning-based algorithms, encapsulated into containers (13). Cytomine can also be installed as a desktop software tool but then without collaboration functionalities. It can also be used through Icy desktop application given the flexibility and openness of these two software tools (14).

The second web tool, called Digital Slide Archive [https://github.com/DigitalSlideArchive, (15), permissive license], was initiated at the University of Atlanta and builds upon the Cancer Slide Digital Archive (see next section). It relies on similar technologies and includes HistomicsTK, a library providing algorithms for image analysis tasks such as color normalization and deconvolution, cell-nuclei segmentation, and positive pixel counting. It is specialized for cancer investigations; hence, its applications are fewer, so far.

### OA Collections for Research or Education Purposes

Until recently, technical challenges due to the large amounts of imaging data generated by slide scanners made it difficult to easily share WSI between remote labs. As a consequence, large data sharing initiatives are still rather few although very promising for both education and research purposes. In the recent years, datasets of significant sizes have been published in the context of image analysis challenges or as companion of research papers. The Camelyon challenge [https://camelyon17.grand-challenge.org/, (16)] used Google Drive to share 1399 H&E-stained sentinel lymph node sections of breast cancer patients for automated detection and classification of breast cancer metastases. Each WSI has slide-level label indicating whether it contains no metastases, macro-metastases, micro-metastases, or isolated tumor cells. A subset of 209 WSIs have detailed hand-drawn contours for all metastases. The PanNuke dataset [https://jgamper.github.io/PanNukeDataset/, (17)] contains 216.4K labeled nuclei from more than 20K WSI at different magnifications. The ANHIR challenge (https://anhir.grand-challenge.org/) for Automatic Non-rigid Histological Image Registration presents hundreds of different types of histopathology tissue (lesions, lung lobes, mammary gland) stained with different dyes and where landmarks have been manually annotated to assess image registration performances.

Other data collections are accessible more easily for end-users through web viewing applications. The Cancer Slide Digital Archive (https://cancer.digitalslidearchive.net/) hosts tens of thousands of WSIs from The Cancer Genome Atlas. The Image Data Repository [(18), https://idr.openmicroscopy.org/tissue/] currently hosts two DP imaging collections. The Cytomine Open Data Collection (https://cytomine.coop/collection) currently offers tens of high-quality WSI of animal and human histology sections originally used in education settings (http://www.histology.be), with an associated license for each image. The University of Leeds Virtual Pathology Project (https://www.virtualpathology.leeds.ac.uk/) hosts more than 385K digital slides, but these are the property of the University of Leeds and software used to visualize them are not OSS.

## Discussion

Without concrete applications, open initiatives would have no practical value in DP. It is remarkable to note that aforementioned OSS and OD collections were successfully used in DP in the recent years to address tens of diverse biomedical research questions or to teach histology and pathology to thousands of biomedical students. QuPath desktop software was used in various fields including oncology and immunology (19), cardiovascular diseases (20), or multiple sclerosis (21) (see other publications at https://github.com/qupath/qupath/wiki/Citing-QuPath). Cytomine web application was used by researchers in various fields including machine/deep learning (22), learning analytics, image analysis in nephrology [e.g., when combined with Icy; (14)], osteoarthritis (23), or lung cancer (24) (see other publications at https://uliege.cytomine.org/#publications). Cytomine was also used in education settings by more than 15,000 users from 85 countries (including developing countries), through a massive open online course (25).

Overall, these tools are being increasingly used in ongoing international research projects worldwide. This broad community of users allows continuous improvement of OSS provided that financial means are set up to guarantee their sustainability beyond the research projects that helped to initiate their development. In order to translate these software tools into production environments (practical pathology courses with thousands of students, or in clinical routine workflows), it is required to provide services (such as training, support, maintenance, specific software developments, etc.) and to rely on pragmatic business development models as these additional efforts could hardly be provided by original authors of these tools. For example, an open, not-for-profit, cooperative company was founded to improve Cytomine software sustainability (http://cytomine.coop). While these kinds of initiatives ease the use of open tools, further efforts should be undertaken to spread their use, particularly in developing countries.

The availability of OD collections also enabled new discoveries and applications; e.g., the Cancer Slide Digital Archive was used to train deep learning models for the classification of adenocarcinoma and squamous cell carcinoma. These models were subsequently applied to independent datasets of FFPE tissues and were also successfully used to directly predict specific mutated genes from pathology images (26). However, there are some limitations with some OD collections in DP. With some of them, there is a lack of precise, semantic, annotations to train deep learning models, and/or a lack of metadata [which can lead to biases if images are reused without precaution; (27)]. In some cases, an associated license that clearly defines the limits of their reuse is missing. Moreover, although these collections are of consistent size, they remain well below what a pathology laboratory deals with routinely, which raises the question of the representativeness of these current collections and derived AI models.

Overall, not all open initiatives have been as successful as we have just presented it. For example, some software packages have hardly been used beyond the laboratory in which they were developed. We believe future community efforts should be undertaken to maximize their reusability. Building a comprehensive, searchable, database of existing tools, such as Bioimage Informatics Search Engine (BISE, http://biii.eu), should be encouraged. In addition, continuous efforts should aim at integrating latest libraries and image analysis packages into collaborative OSS platform presented herein to avoid duplication of efforts. These collaborative software tools might also be further enhanced to disseminate data collections with detailed annotations and metadata and therefore enable the organization of high-impact studies or challenges. Indeed, we believe open and collaborative principles should be further followed to accelerate scientific progress and hence societal impact. In our opinion, individualist practices (where imaging datasets, algorithms, quantification results, and associated knowledge are still often stored and analyzed within the restricted circle of a specific laboratory) should be surpassed. Overall, open initiatives highlighted in this paper might pave the way to more collaborative DP in the future.

## Author Contributions

The author confirms being the sole contributor of this work and has approved it for publication.

### Conflict of Interest

RM is co-founder of the not-for-profit Cytomine SCRL FS (cooperative company with social purpose).
